# Development and feasibility testing of a mobile phone application to track children’s developmental progression

**DOI:** 10.1371/journal.pone.0254621

**Published:** 2021-07-15

**Authors:** Patricia Kitsao-Wekulo, Nelson Kipkoech Langat, Margaret Nampijja, Elizabeth Mwaniki, Kenneth Okelo, Elizabeth Kimani-Murage

**Affiliations:** 1 Maternal and Child Wellbeing Unit, African Population and Health Research Center, Nairobi, Kenya; 2 Data, Measurement and Evaluation Unit, African Population and Health Research Center, Nairobi, Kenya; Columbia University, UNITED STATES

## Abstract

Given that mobile phone usage has increased rapidly throughout the world, one possibility to increase parental involvement in monitoring their children’s progression is to train parents or primary caregivers on the use of mobile phone technology to track their children’s developmental milestones. The current paper aimed to describe the development of a mobile phone application for use among primary caregivers and establish the feasibility and preliminary impact of caregivers using a mobile phone application to track the progression of their children’s development in a context where there is a paucity of similar studies. This study is a substudy that focusses on the intervention group only of a recently completed two-armed quasi-experimental study in an informal settlement in Nairobi. The mobile phone application which consisted of questions on children’s developmental progression, as well as stimulation messages, was developed through a step-wise approach. The questions covered five child developmental domains: communication; fine motor; gross motor; personal-social; and, problem-solving. Depending on the response received, the child would be classified as having ‘achieved a milestone’ or ‘milestone not achieved.’ If a child had achieved the milestone for a specific age, a caregiver would receive an SMS on how to stimulate the child to achieve the next milestone. Where the milestone was not achieved, the caregiver would get a message to enhance development in the area of delay. Caregivers with children aged between six months and two years were recruited into the study and received questions and messages regarding their children’s development (age-specific) on a monthly basis for 12 months. Caregiver adherence to the intervention was above 90% in the first three months of implementation. Thereafter, the response rate fluctuated between 76% and 86% across the subsequent months of the intervention. The high level and fairly stable caregivers’ rate of response to the 12 rounds of messaging indicated feasibility of the mobile technology. Further, in the first three months of intervention implementation, the majority of caregivers were able to keep track of how their children attained their developmental milestones. The intervention seems to be scalable, practical and potentially low-cost because of the wide coverage of phones.

## Introduction

Lack of bonding with the caregiver and inadequate stimulation of young children have been associated with sub-optimal developmental outcomes, particularly during the first 1,000 days when brain development is most rapid [1]. On the other hand, appropriate stimulation during the early years is linked to better physical, emotional and cognitive development, and with higher academic achievement in later years [2]. However, primary caregivers in low- and middle-income countries (LMICs) may often not be aware of the causal link between their behaviors and their children’s development [3]. This situation may be exacerbated by economic deprivation which further decreases the likelihood that parents are able to provide adequate and appropriate stimulation to their young children [4].

Interventions that seek to improve parenting behavior are considered one of the ways in which the quality and quantity of interactions between a primary caregiver and a young child can be enhanced. This is so because responsive parenting is a modifiable behavior that can positively influence child developmental outcomes. Given that mobile phone usage has increased rapidly throughout the world, one possibility to increase parental involvement in monitoring their children’s progression is to train parents or primary caregivers on the use of mobile technology to track their children’s developmental milestones. This is in light of earlier reports which suggest that high levels of parental involvement increase the likelihood that an intervention will be effective [5]. Moreover, enabling primary caregivers to track their children’s development facilitates the timely identification of developmental delays. This is more so in disadvantaged settings where young primary caregivers may lack the requisite knowledge to make appropriate childcare decisions [6]. As children’s development takes place through daily life activities, primary caregivers should be able to report early signs of developmental delay during their regular interactions with children. Further, to boost the effectiveness of such interventions, engagement of community health volunteers (CHVs) who are likely to have an intimate relationship with those they serve within their communities [7], could go a long way in facilitating behavior change among primary caregivers.

Certain functional and structural qualities that make mobile phone technology attractive as a health-related intervention are that it: 1) is a low-cost channel that is user-friendly and wide reaching; 2) provides instantaneous access and direct communication which allow for faster transfer of information; and, 3) increases the ability of researchers to tailor interventions to participants in real time [8–11]. Moreover, the low bandwidth requirements of applications such as text messaging promote the wide use of mobile phone technology. For this reason, mobile phone interventions, ranging from simple text messages to complicated applications, can be used either as a one-way system where the participants receive suggestions for behavior or allow tailored feedback based on data received [12]. In past studies, they have been found to be effective in changing health behaviors in a positive way. For instance, mobile health interventions have been used to track children’s immunization visits, reduce dropouts for vaccinations, improve attendance to clinic visits, promote changes in physical activity, prevent, treat and control chronic illnesses and monitor and improve dietary behavior [8, 13–21]. Given its wide use in health-related interventions, it would be worthwhile to harness the potential of mobile phone technology use in other areas.

Although there are several studies reporting the use of mobile phone technology to track a range of health and behavioral outcomes, there are hardly any reports on its application among parents to monitor developmental progression of very young children [5]. Moreover, few studies have used mobile phone technology specifically to train parents or caregivers on how to monitor their children’s developmental progress. This is particularly so in sub-Saharan Africa (SSA) where the vulnerability status of a specific population may render them hard to reach and difficult to work with. Further, the needs of vulnerable populations may vary as they may face unique challenges.

Based on data for Nairobi City County, we extrapolated that more than 80% of the population in informal settlements has access to a mobile phone through individual ownership or shared use [22]. The use of mobile phone technology therefore offered an innovative opportunity to meaningfully engage with highly disadvantaged populations in tracking and monitoring their children’s development. The current paper is based on a study that sought to test the feasibility of using a mobile phone application to track children’s developmental progress in an informal settlement in Nairobi [23]. Our aims were to: a) describe the development of a mobile phone application for use among primary caregivers; and b) establish the feasibility and preliminary impact of caregivers’ use of a mobile phone application to track the progression of their children’s development. The current paper provides valuable information on the development and feasibility of the use of mobile phone technology to track progression in a context where there is a paucity of similar studies.

## Methods

### Development of the intervention

The research team from the African Population and Health Research Center (APHRC) in partnership with Val Partners (the consultant that managed the mobile technology platform and provided back-end support for the project) developed an integrated early childhood development (ECD) mobile phone application to help young mothers respond to their children’s developmental progress in a timely manner. In the co-design phase, appropriate child stimulation messages were created through two focus group discussions (FGDs), each with 8–10 caregivers, to establish the type of messages to be delivered based on current caregiving practices, and social and cultural contexts. In brief, some of the findings that emerged through these interviews illustrated that there were gaps in the way caregivers engaged with young children. For instance, some caregivers noted that children in the community played by themselves most of the time as they (caregivers) were away at work for an extended period of time during the day. And, when these caregivers got back home, they were too tired to engage in any developmentally appropriate activities with their children. Caregivers also tended to use directive language in their communication with young children. These findings suggested the need to focus the messages on areas in which caregivers could engage with their children in a developmentally-appropriate manner. In the second step, the information generated was used to develop a health messaging platform. The platform included questions for caregivers on their children’s development across the various domains, and the stimulation messages that would be sent to them which were dependent on their responses (Yes or No). Some examples of these questions and stimulation messages are provided in Table 1. In the third step, community health volunteers (CHVs) (N = 12) and caregivers were trained over a period of five days, and mentored on the use of the mobile phone technology to record and track achievement of milestones and to identify key development delays. The mobile phone technology innovation was capable of generating customized child stimulation messages based on the age of the child and previous feedback.

**Table 1:** Examples of questions and stimulation messages.

Domain/ Question/ (Age)	Stimulation message if response is ‘Yes’	Stimulation message if response is ‘No’
***Communication***		
When a sound is made, does your child turn and look in the direction of the sound? (7 months)	Continue speaking with your child or making sounds using different objects in your immediate environment	Produce a sound using different objects within your immediate environment and encourage your child to turn towards the sound
***Gross motor***		
Is your child able to walk along a table while supporting him/herself with one hand? (12 months)	Continue to provide opportunities for your child to walk while supporting him/herself with one hand	Place your child in a standing position next to a table or chair. Stay close to him/her while encouraging him/her to come towards you
***Fine motor***		
Is your child able to throw a small ball towards you when you’re standing in front of him/her? (15 months)	Continue to provide opportunities for your child to throw a ball forwards	Show your child how to throw a ball forwards
***Problem Solving***		
Does your child imitate you after watching you draw a line from the top to the bottom of a paper using a pencil? (22 months)	Continue showing your child how to draw on a paper using a pencil. Provide for him/her paper and pencil to draw on his/her own	Show your child how to draw on a paper and give him/her an opportunity to do so
***Personal Social***		
Does your child drink from a cup or a glass, and return it to the table with little spillage? (22 months)	Encourage your child to continue drinking from a cup or glass without spilling	Show your child how to drink from a cup or a glass. Provide him/her with opportunities to drink using a cup or glass

#### Application of the intervention

The tracking messages were sent to caregivers every month. The caregivers were then required to respond to the questions received. The questions covered five developmental domains: communication; fine motor; gross motor; personal-social; and, problem-solving. Depending on the response received, the child would be classified as having ‘achieved a milestone’ or ‘milestone not achieved.’ Caregivers whose children had achieved the milestone for a specific age received a short message service (SMS) on how to stimulate the child to achieve the next milestone. In the case where a child had not achieved a certain milestone, the caregiver would get a message to enhance development in the area of delay. In addition, this was flagged on the mobile phone application, and a message was sent to the assigned CHV for action. The CHV then visited the household to provide the necessary support to the caregiver and to establish means in which to address the delay. The CHVs also reminded caregivers to respond to the messages in the case where no response had been provided in a specific month. Each CHV was assigned to follow up between seven and 13 caregivers. Mentorship visits by CHVs and referral/linkages to the health facility were expected to be used as a means to address the identified developmental delays.

**Fig 1:**
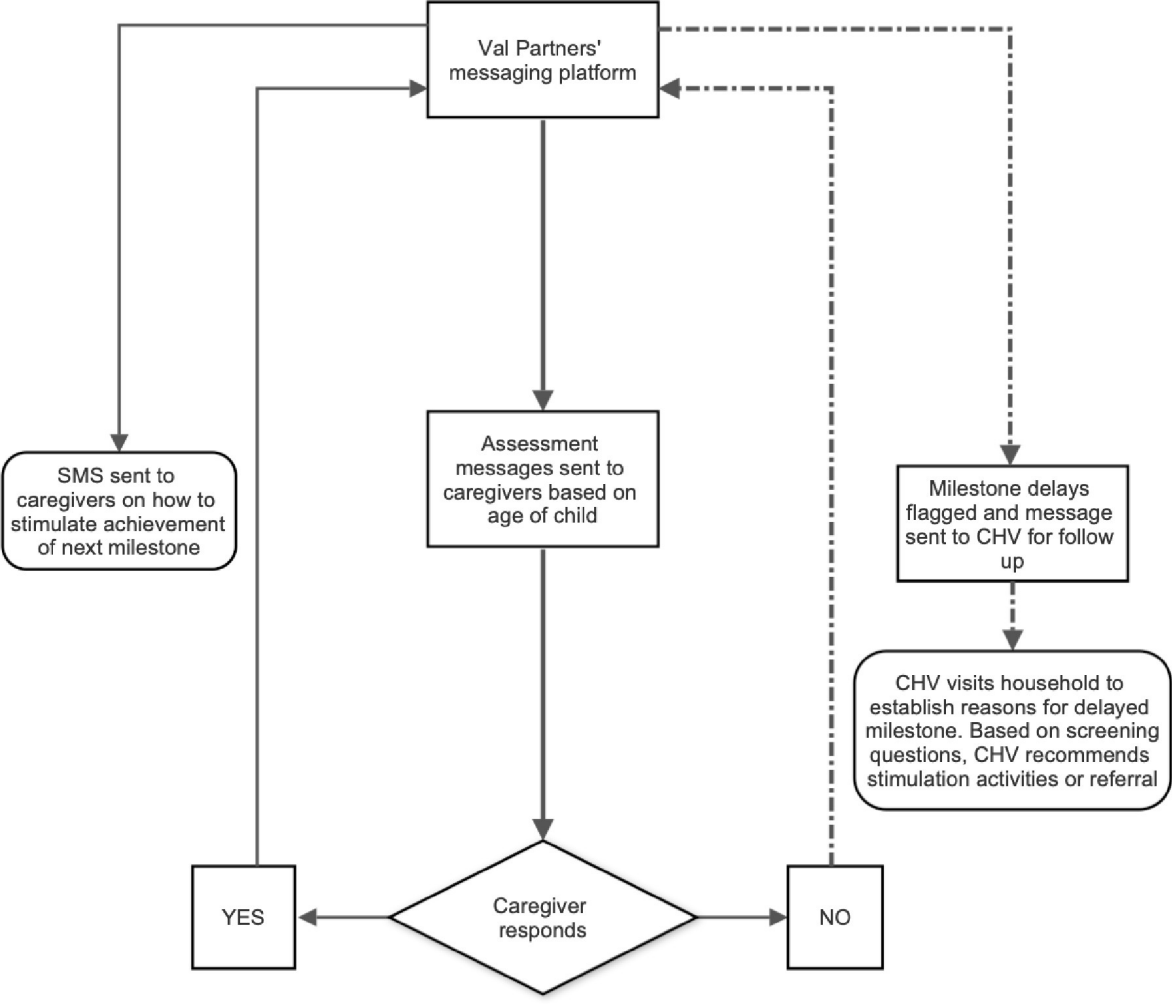
Flow chart showing implementation of the messaging process.

The study and implementation teams held monthly monitoring and evaluation meetings with the CHVs to assess the performance of the SMS platform and how the caregivers were responding. The monthly meetings served as a forum: a) for feedback from the CHVs on how the caregivers operationalized the messages they received through the system; b) to enable the team to troubleshoot any challenges faced by caregivers who did not respond in time or at all; and, c) to record various lessons for future implementation. The intervention was developed using a stepwise approach and worked as shown in Fig 1.

### Feasibility testing

#### Study design

The current paper is based on a cross-sectionally designed substudy of a recently-completed two-armed quasi-experimental study. The substudy focused only on primary caregivers in the intervention group, as well as the CHVs who supported them. In this group (intervention), caregivers with children aged between six months and two years were recruited into the study and an SMS sent to them regarding their children’s development (age-specific) monthly for 12 months. The primary outcome was the feasibility of the intervention among primary caregivers.

#### Study site and participants

The substudy was conducted in Korogocho Ward within Ruaraka sub-County, Nairobi. Korogocho is one of the largest informal settlements in Nairobi. We chose this site as anecdotal reports suggested that many of the caregivers in this area were young mothers aged between 15 and 19 years who were likely to have limited childcare knowledge.

**Fig 2:**
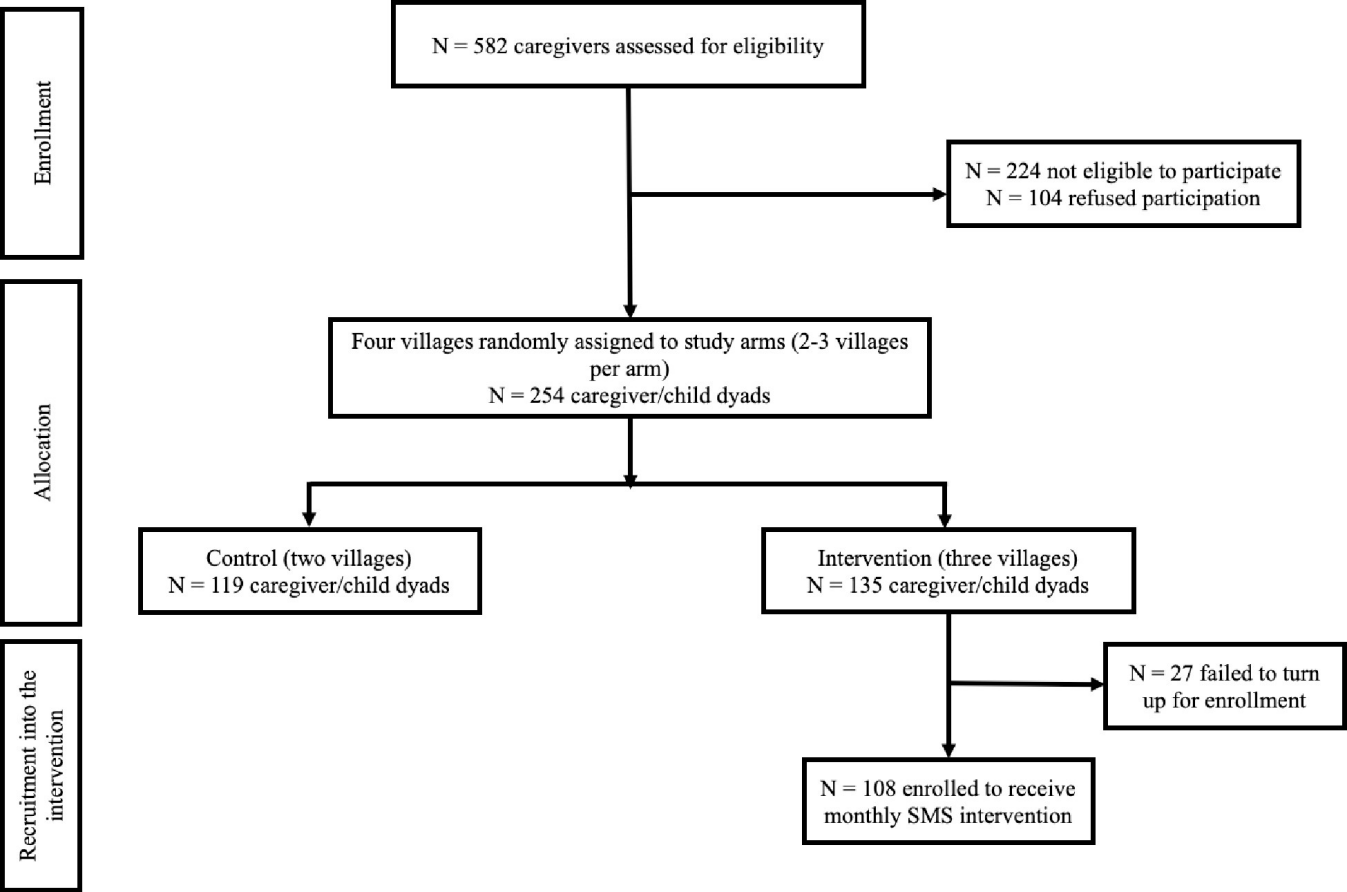
Flow diagram of the recruitment of participants into the main study (this paper focusses on the intervention arm only).

Participants were primary caregivers of children aged between six and 24 months. The recruitment process is illustrated in Fig 2. Potential participants were identified from the Nairobi Urban Health and Demographic Surveillance System (NUHDSS) database which is run by the APHRC, and assessed for eligibility (N = 582). A total of 224 participants did not meet all the eligibility criteria while 104 refused to participate in the study for various reasons such as lack of permission from their partners, lack of time and no interest in the study. Families were selected on the basis of their vulnerability status (for example, teenage mothers, poor health and nutrition indicators, HIV exposure, and poverty levels). Additionally, they were required to be long-term residents (more than one year) of the study area as the intervention was planned for 12 months. The final study sample had 254 primary caregiver/child dyads of whom 119 were allocated to the comparison arm and 135 to the intervention. We over-recruited the sample as we were aware that phone ownership in informal settlements is likely to be influenced by gender, education and age, which suggested that females, those who were less educated, and those who were young were less likely to own phones [24]. Of the 135 caregiver/child dyads allocated to the intervention, a total of 108 were enrolled into the intervention after 27 failed to turn up during enrollment. These 108 caregiver/child dyads formed the sample for the study reported in this paper, since the main aim was to test the feasibility of the intervention.

#### Data collection tools and procedures

At baseline, trained data collectors administered the Ages and Stages Questionnaire–Third Edition (ASQ-3) [25] to establish children’s ‘developmental’ age before the start of the intervention. The ASQ-3 was used to measure children’s developmental outcomes in five domains (gross and fine motor skills, communication, personal-social, and problem-solving) through a combination of primary caregiver self-reported questions and direct observations [26]. Information on developmental age enabled us to determine the level at which the messages that would be sent to the mothers/caregivers should begin, as the messages on the mobile phone application were crafted according to the age of the child. For instance, with regards to motor development, if the mother reported that the child was able to ‘sit on his/her own,’ the messages that would be sent to the mother/caregiver at the first contact were those where the mother was encouraged to stimulate the development of the next milestone, that is, crawling. Thereafter, 108 caregivers were recruited into the SMS system where they received monthly questions to which they provide “Yes” or “No” responses depending on their children’s milestone achievement. Responses from 102 participants were available for analysis. These monthly data together with the baseline data formed the basis for the analysis carried out for the current paper.

#### Data management and analysis

Data cleaning, coding and analysis were done using Stata version 15. Descriptive analysis was carried out to explore the data. Categorical variables (e.g. education level) were summarized using frequencies and percentages while continuous variables (e.g. age) were summarized using means (standard deviation: SD) or medians (interquartile range: IQR) depending on the nature of the distribution. Trends in adherence to the intervention were illustrated in a table.

#### Ethical considerations

The study was conducted after internal review by the APHRC’s Ethics Review Committee. Ethics approval (Approval number– P578-2018) was provided by the Amref Health Africa’s Ethics and Scientific Review Committee (ESRC). We obtained permission to conduct the study from the National Commission for Science, Technology and Innovation (NACOSTI). We sought informed consent from all study participants who were required to provide a signature on the consent form. Primary caregivers who were below the age of 18 years but above the age of 15 years were considered as ‘mature minors’ (as classified by the ESRC) and provided consent on their own behalf. We asked those who could not read or write to provide a thumbprint to signify consent, and this was done in the presence of a witness.

## Results

### Socio-demographic characteristics

More than half (56.9%) of the caregivers had attained primary school level education. About three quarters (71.6%) were married and nine out of ten (89.2%) primary caregivers were the mothers of the children. Over half (60.8%) of the primary caregivers lived in households with four to six members. Slightly more than 40% of the households were classified as falling into the lowest wealth tertile. The majority of caregivers were aged below 30 years, with a median (IQR) age of 28.2 (21.9–32.8) years. Children’s ages ranged from seven to 24 months with a median of 18 months (Table 2).

**Table 2:** Socio-demographic characteristics of primary caregivers and children, N = 102.

	Frequency (n)	Percent (%)
**Child sex**		
Male	46	45.1
Female	56	54.9
**Caregiver education**		
Primary	58	56.9
Secondary	39	38.2
Tertiary	5	4.9
**Marital status**		
Single	19	18.6
Married	73	71.6
Divorced/Separated/Widowed	10	9.8
**Relationship to child**		
Mother	91	89.2
Father	3	2.9
Other	8	7.8
**Household size**		
< = 3 members	18	17.6
members	62	60.8
>6 members	22	21.6
**Wealth index tertile**		
Low	42	41.2
Middle	29	28.4
High	31	30.4
	**Median**	**IQR**
Caregiver age (years)	28.2	21.9–32.8
Child age (months)	18.0	12.8–22.3

### Response rates of caregivers during the intervention period

Caregivers began to receive the SMSs asking questions regarding their children’s development a month after they were enrolled into the study, and thereafter on a monthly basis for 12 months. Table 3 illustrates that the adherence rate was at the highest level in the first three months of the intervention period. Out of 108 caregivers who received questions about their children’s developmental progression, 103 (95%) responded to the SMSs in the first month of the intervention. In each of the second and third months, 102 (94%) responded to the SMSs. Thereafter, the response rate dropped slightly and fluctuated between 75% and 86% across the subsequent months. Notably, there was a significant drop in the response rates in October 2019 (94.4% to 86.1%) and April 2020 (83.3% to 76.9%) compared to the previous months.

**Table 3:** Proportion of caregivers who reported on their children’s developmental milestones (N = 108).

Month	Total number of caregivers that responded	% response
July	103	95.4
August	102	94.4
September	102	94.4
October	93	86.1
November	90	83.3
December	87	80.6
January	88	81.5
February	94	87.0
March	90	83.3
April	83	76.9
May	81	75.0
June	87	80.6

### Pattern of children’s developmental progression in the first three months

**Fig 3:**
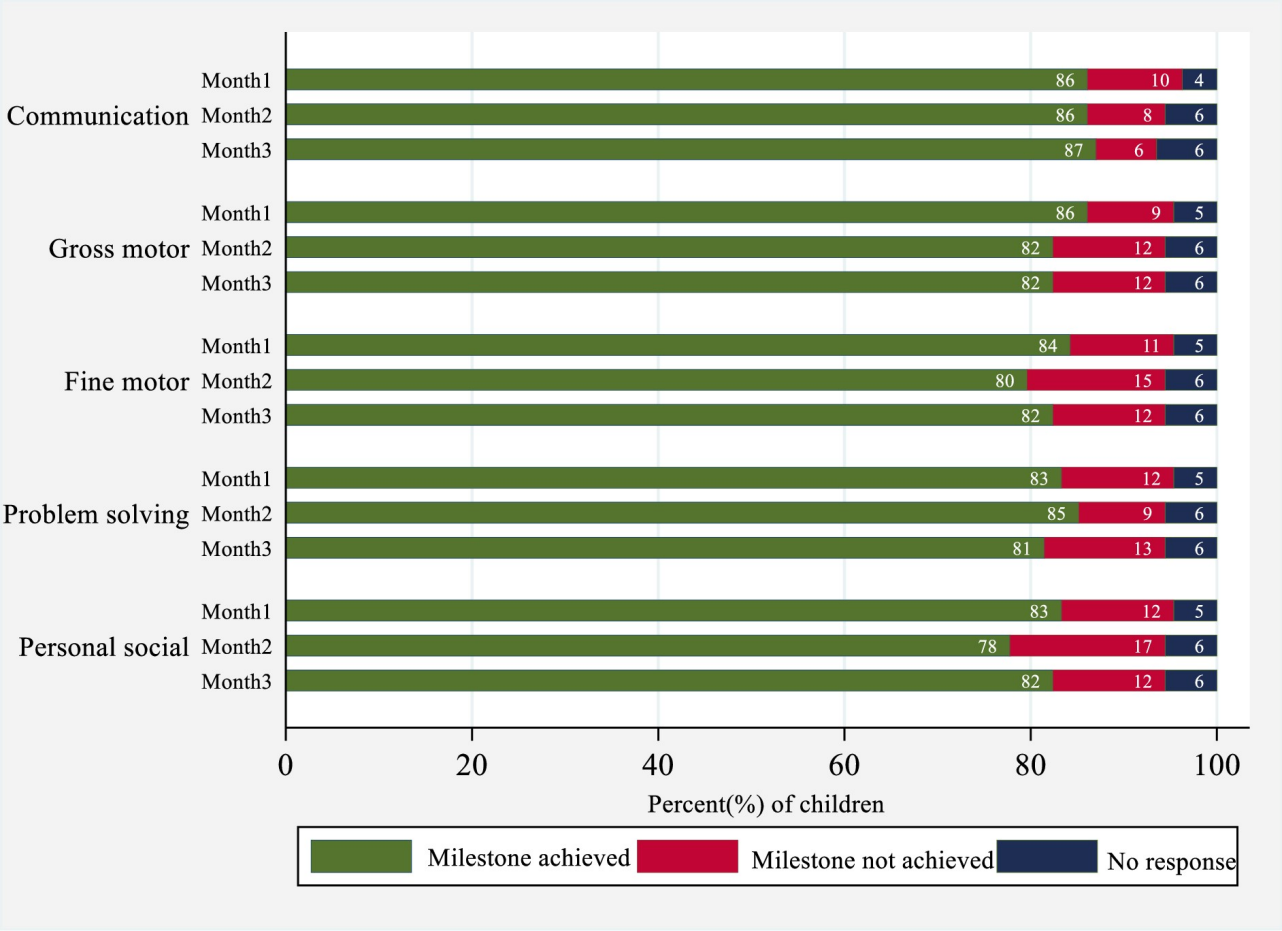
Caregiver report of achievement of developmental milestones by domain across the first three months of intervention.

There was a varied pattern in the achievement of milestones by children as reported by primary caregivers (Fig 3). Across the first three months of the intervention, the proportion of children who achieved communication milestones increased slightly, from 86% in the first month to 87% in the third month (p = 0.798). On the other hand, the proportion of children who achieved gross motor milestones decreased from 86% in the first month to 82% in the third month (p = 0.396). The pattern of milestone achievement for fine motor skills, problem-solving skills and personal-social skills showed fluctuations across the three months. In each domain, there was no significant difference in the proportion of children who achieved the milestones between the first month and the third. About 6% of caregivers did not provide a response on their children’s developmental status across this early intervention period.

### CHV adherence to the intervention protocol

On average, CHVs made phone calls to caregivers at least twice a month, and visited the caregivers’ homes once a month to provide mentorship support. In one or two cases, CHVs were not able to establish contact with caregivers even after three follow-up visits. In such cases, caregivers were considered to have dropped out of the study.

## Discussion and conclusion

The current paper aimed to describe the development of, and establish the feasibility of mobile phone technology to track children’s developmental progression. The reported results are based on a cross-sectional substudy involving caregivers in the intervention arm of a recently-completed quasi-experimental study. Providing such data has relevance particularly for the implementation of novel studies, as reports on the use of mobile phone applications that are timed to the age and developmental stage of an infant are rare.

In general, there were generally good response rates to the monthly SMSs. Our findings showed that across the 12 months of the intervention, there was high adherence to the mobile phone technology use among caregivers despite their vulnerability status. In particular, adherence in the first three months was above 90%. This finding suggested that in the early stages, the use of mobile phone technology to track children’s developmental progress was feasible for use among caregivers as a high proportion of them provided responses on their children’s developmental status. Our findings corroborated those of earlier studies [15, 27] and generally, caregivers seemed to be positive about being involved in tracking their children’s outcomes.

When we looked at the monthly pattern, the sharp reduction in the rate of responses in October may have been because some caregivers had become fatigued and were no longer interested in responding to the SMSs. This could be attributed to some caregivers getting accustomed to the intervention and thus lacking the motivation to respond. The drop in response rates in April could be explained by the effects of the COVID-19 pandemic. Some caregivers lost their sources of income which may have affected their overall wellbeing. In such a state, they would be less likely to give attention to responding to the SMSs since they spent most of their time looking for other sources of income. These reductions suggested the need to take into account specific circumstances that caregivers may face during an intervention period, with considerations for actions to take to counter such a situation.

Whereas we recruited 108 caregivers into the intervention, and these were the ones who indicated availability and willingness to participate, the number that actually participated in the first three months was 102. The six caregivers who were excluded could have been willing but were not able to participate because maybe they shared phones with their partners, or did not have access to phones. One of the most important effects of the lack of a phone or phone access was inability to enroll in an intervention that required phone ownership or access. Earlier studies have also shown that although mobile phones have the potential to increase access to health and other information, the gender gap in phone ownership may preclude women’s participation in digital health interventions [28].

The data show that in the first three months of intervention implementation, the majority of caregivers were able to keep track of how their children attained their developmental milestones. The slight differences in the proportions across the different domains were likely to be a reflection of the natural trajectories in achievement of different skills. Development is not linear and does not occur at the same time/ speed for every child and across all the domains.

The intervention seemed to be a scalable, practical and potentially low-cost intervention because of the wide distribution of phones. The high level and fairly stable caregivers’ rate of response across the 12 rounds of messaging indicated feasibility of the mobile technology. Like in any other developing country, mobile phone use in Kenya has increased considerably and many families now own mobile phones. The massive use of technology can therefore be leveraged to help facilitate children’s growth and development, as well as caregivers’ knowledge and practices.

Based on the findings of our study, we suggest the following considerations:

- Mobile phone technology use in tracking children’s developmental progression could enhance the achievement of SDG 4.2 as caregivers are made more aware of the need to monitor their children’s milestone achievement;
- When designing interventions based on mobile phone technology, one needs to take into consideration the fact that when participants report phone ownership, the phone may not necessarily belong to them, or it may be a shared resource;
- Other formats of mobile phone applications should be considered for use with those who may not be able to read, or who may have challenges in navigating the technology.
